# MUSCLE MITOCHONDRIAL ENERGETICS ARE ASSOCIATED WITH GREATER MULTIMORBIDITY IN MEN

**DOI:** 10.1093/geroni/igac059.2915

**Published:** 2022-12-20

**Authors:** Theresa Mau, Terri Blackwell, Anne Newman, Peggy Cawthon, Steve Cummings, Philip Kramer, Russell T Hepple, Stephen Kritchevsky

**Affiliations:** San Francisco Coordinating Center, San Francisco, California, United States; California Pacific Medical Center, San Francisco, California, United States; University of Pittsburgh, Pittsburgh, Pennsylvania, United States; University of California, San Francisco, San Francisco, California, United States; San Francisco Coordinating Center, San Francisco, California, United States; Wake Forest University School of Medicine, Winston-Salem, North Carolina, United States; University of Florida, Gainesville, Florida, United States; Wake Forest School of Medicine, Winston Salem, North Carolina, United States

## Abstract

Older adults who have poorer muscle mitochondrial energetics tend to have lower cardiorespiratory fitness—which is associated with the risk of cardiovascular disease, cancer, and dementia. In the Study of Muscle, Mobility, and Aging (SOMMA), we tested the associations of muscle mitochondrial energetics (MME) with a multimorbidity index in this cohort of women (Nf507) and men (Nf349) 70+ years of age. The index included 13 age-related chronic conditions (arthritis, cancer, cardiac arrhythmia, chronic kidney disease, chronic obstructive pulmonary disease, coronary artery disease, congestive heart failure, dementia, depression, diabetes, osteoporosis, stroke, and aortic stenosis). Participants were grouped by 0, 1, 2, or 3+ conditions and were on average 76.3 years old, 59.2% women, 12.5% Black, 85.5% White, with 2.0% reporting other identities. MME were measured in permeabilized muscle fibers by high-resolution respirometry in vitro (Leak and MaxOXPHOS) and in vivo by 31P-magnetic resonance spectroscopy (ATPmax). The gender interaction p-value with MME was 0.04 (Leak), 0.34(MaxOXPHOS), and 0.09(ATPmax). In men, we found that for every 1 SD decrease of mitochondrial respiration, there was a significant increase of proportional odds ratio (POR) for higher multimorbidity count: POR (95%CI) for Leak was 1.47(1.18, 1.86), p < 0.001 and for Max OXPHOSCI+CII was 1.35(1.08,1.68), p < 0.01. For ATPmax, was 1.22(0.98,1.52), p=0.08. These were adjusted for age, race, site, education, smoking status, weight, height, weight*height, and self-reported physical activity. These trends were absent in women (Leak=0.97(0.79, 1.20), p=0.79, MaxOXPHOS=1.05(0.86, 1.28), p=0.66, ATPmax=0.98 (0.82, 1.17), p=0.81), and future analyses will deconstruct the multimorbidity index to determine gender-specific associations with each condition.

